# Effect of Dietary Chlorogenic Acid on Growth Performance, Antioxidant Function, and Immune Response of Broiler Breeders under Immune Stress and Stocking Density Stress

**DOI:** 10.3390/vetsci9100582

**Published:** 2022-10-21

**Authors:** Dongying Bai, Kexin Liu, Xianglong He, Haiqiu Tan, Yanhao Liu, Yuqian Li, Yi Zhang, Wenrui Zhen, Cai Zhang, Yanbo Ma

**Affiliations:** Henan International Joint Laboratory of Animal Welfare and Health Breeding, College of Animal Science and Technology, Henan University of Science and Technology, Luoyang 471023, China

**Keywords:** stress, chlorogenic acid, growth performance, antioxidant, anti-inflammatory

## Abstract

**Simple Summary:**

Immune stress and high stocking density stress are two major concerns in poultry production, as they can greatly impair the productive and reproductive performance of chickens with serious economic consequences. Chlorogenic acid has been widely used as a feed additive in poultry production due to its potent antioxidant, anti-inflammatory, antibacterial, and antiviral activities. This study aimed to investigate the effects of dietary chlorogenic acid supplementation on growth performance, antioxidant function, and immune response in broiler breeders exposed to immune stress and high stocking density stress. Our study highlights that chlorogenic acid increased feed intake, downregulated serum corticosterone levels, and altered the immune and antioxidant functions of broiler breeders exposed to immune stress or high stocking density stress. Dietary inclusion of 1 g/kg chlorogenic acid could be used to increase feed intake of broiler breeders and alleviate the effects of immune stress and exposure to high stocking density in poultry.

**Abstract:**

The study was conducted to evaluate the effects of dietary chlorogenic acid supplementation on the growth performance, antioxidant function, and immune response of broiler breeders exposed to immune stress or high stocking density stress. The test was divided into two stress models. For the immune stress test, 198 birds were distributed into three experimental treatments with six replicates per treatment. The treatments were: (1) saline control (birds injected with saline and fed basal diet), (2) LPS group (birds injected with 0.5 mg LPS/kg body weight and fed basal diet), and (3) CGA + LPS group (birds injected with LPS and fed basal diet supplemented with 1 g/kg CGA. LPS was intraperitoneally injected from day 14, and then daily for 10 days. For the high stocking density stress model, 174 birds were distributed into three experimental treatments with six replicates per treatment. The treatments were: (1) controls (birds fed basal diet and raised at a stocking density of 14 broilers per m^2^), (2) high-density group (birds fed with basal diet and raised at a stocking density of 22 broilers per m^2^), and (3) high density + CGA group (birds fed with 1 g/kg CGA and raised at a stocking density of 22 broilers per m^2^). Results showed that LPS injection and high stocking density significantly decreased the body weight and feed intake of broiler breeders, while CGA supplementation increased feed intake of broiler breeders under LPS injection and high stocking density stress. Moreover, LPS injection and high stocking density increased the concentration of corticosterone in serum, and CGA addition remarkably downregulated serum corticosterone levels. The GSH level decreased with LPS injection and CGA increased the GSH concentration in the intestines of immune-stressed broiler breeders. LPS injection promoted the production of circulating proinflammatory cytokines (serum IL-1β and TNF-α) by 72 h after LPS injection. Dietary supplementation with CGA prevented the increase in serum TNF-α caused by LPS. These results suggest that dietary inclusion of 1 g/kg CGA could increase the feed intake of broiler breeders and alleviate the effects of inflammatory mediator stress and exposure to high stocking density.

## 1. Introduction

Stressors can affect the central nervous system, activate the hypothalamic-pituitary-adrenal axis, stimulate the receptors of signal molecules related to appetite, and enhance the synthesis and secretion of stress hormones such as corticosterone, resulting in increased catabolism and anorexia. In addition, stressors can stimulate macrophages to produce inflammatory cytokines such as interleukin-1 (IL-1) and tumor necrosis factor-α (TNF-α), which can affect peripheral tissues and the central nervous system to alter the body’s metabolism [1,2]. Immune stress and high stocking density stress are two major concerns in poultry production, as they can greatly impair the productive and reproductive performance of chickens with serious economic consequences [3,4,5,6,7]. Thus, there is an urgent need to explore the mechanism of immune stress and high stocking density stress on poultry health, in order to develop effective mitigation strategies to reduce subsequent production loss.

Chlorogenic acid (CGA) is one of the most abundant phenolic acids in nature [8]. It is widespread in many plants and Chinese herbal medicines [9]. Phenolic acids have been widely used as feed additives in poultry production due to their potent antioxidant [10,11,12], anti-inflammatory [13,14,15], antibacterial [16,17], and antiviral [18,19] activities. Although reports concerning the usefulness of CGA in poultry production have steadily increased, there is still little known about its ability to modulate the immune response, antioxidant function, and growth performance of broiler breeders under immune stress and high stocking density stress. Therefore, this study aimed to investigate the effects of dietary CGA (purity 98%) supplementation on growth performance, antioxidant function, and immune response in broiler breeders exposed to immune stress and high stocking density stress.

## 2. Materials and Methods

A total of 372 one-day-old male Arbor Acres broiler breeders obtained from a commercial hatchery (Henan Quanda Poultry Breeding Company, Hebi, China) were used to assess the effect of dietary CGA (purity 98%, Changsha Staherb natural ingredients Co., Changsha, China) on the growth performance, immune response, and antioxidant function of broilers under immune stress and stocking density stress. One hundred and ninety-eight birds were randomly assigned to three experimental treatments with six replicates per treatment. For the immune stress test, the three treatments were: (1) saline controls (birds injected with saline and fed basal diet), (2) LPS group (birds injected with 0.5 mg of LPS/kg body wgt and fed basal diet), and (3) CGA + LPS group (birds injected with LPS and fed basal diet supplemented with 1 g/kg CGA. Birds in the immune stress test were raised at a stocking density of 14 broilers per m^2^. CGA was added throughout the trial period and LPS was intraperitoneally injected from day 14, and daily for 10 days. One hundred and seventy-four birds were distributed to three experimental treatments with six replicates per treatment. For the stocking density stress test, the treatments were: (1) control group (birds fed basal diet and raised at a stocking density of 14 broilers per m^2^), (2) high-density stocking group (birds fed basal diet and raised at a stocking density of 22 broilers per m^2^), and (3) high-density stocking + CGA group (birds fed with 1 g/kg CGA and raised at a stocking density of 22 broilers per m^2^). A corn-soybean meal basal diet was formulated to meet or exceed NRC (1994; Table 1) without any additives except for an anti-coccidial drug. Broilers were housed in an environmentally controlled chamber with feed and water ad libitum. All birds in each of the six treatment groups received vaccinations for infectious bronchitis virus and Newcastle disease virus on day 1 (d1) and d20, and bursal disease virus on d14.

Birds under immune stress were weighed and the feed intake (FI) and body weight (BW) were recorded on days 14, 15, 17, 19, 21, and 23. The average daily weight gain, average daily feed intake, and feed conversion rate (FCR) of each group were calculated. Six birds per treatment under immune stress were sampled on days 14, 15, 17, 19, 21 and 23. Additional 14-day samples were taken at 2 h and 4 h after LPS injection. Birds under stocking density stress were weighed and the feed intake and body weight were recorded on days 7, 14, 21, and 28. The average daily weight gain, average daily feed intake, and FCR of each group were calculated. Six birds under stocking density stress per treatment were sampled on days 14, 21, and 28. Blood was drawn from the wing veins of six randomly chosen birds, allowed to clot, and serum obtained by centrifugation. Sampled birds were euthanized by cervical dislocation and two-cm-long intestinal segments (mid-duodenum, mid-jejunum and mid-ileum) were removed, snap-frozen in liquid nitrogen, and stored at −80°C. The concentrations of cortisone (CORT), IL-1β, TNF-α, glutathione (GSH), and malondialdehyde (MDA) were determined with commercial kits according to the manufacturer’s instructions (Jian Cheng Bioengineering Institute, Nanjing, China).

The normality of all data was checked, and data analysis was performed with SPSS statistical software (ver. 20.0 for Windows, SPSS Inc., Chicago, IL, USA) by using one-way ANOVA followed by Duncan’s multiple comparison tests. Significance was considered as *p* < 0.05.

## 3. Results

### 3.1. Growth Performance

**Figure 1:**
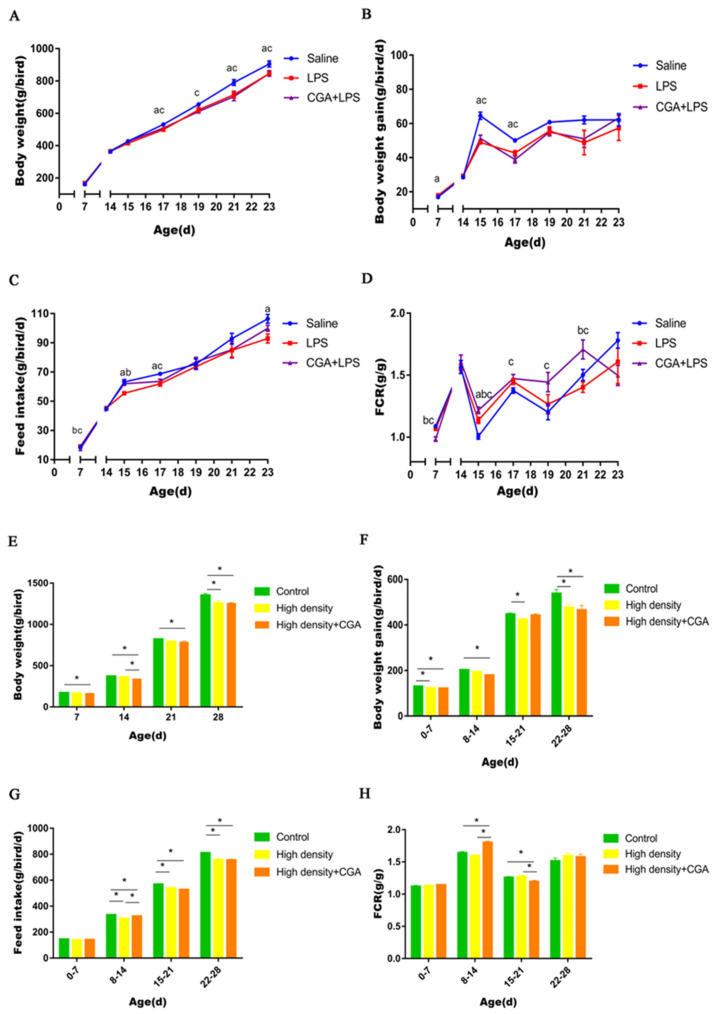
Effects of chlorogenic acid on broiler performance challenged with immune stress and high density stress. (**A**) Body weight of broilers challenged with immune stress. (**B**) Body weight gain of broilers challenged with immune stress. (**C**) Feed intake of broilers challenged with immune stress. (**D**) Feed conversion ratio of broilers challenged with immune stress. (**E**) Body weight of broilers challenged with high density stress. (**F**) Body weight gain of broilers challenged with high density stress. (**G**) Feed intake of broilers challenged with high density stress. (**H**) Feed conversion ratio of broilers challenged with high density stress. a means significant difference between Saline and LPS groups, b means significant difference between LPS and CGA + LPS groups, c means significant difference between Saline and CGA + LPS groups, and * means significant difference between groups.

LPS injection significantly decreased body weight on days 17, 21, and 23 in contrast to control (*p* < 0.05). The body weight of broilers in the saline control group was higher than in the CGA + LPS group on days 17, 19, 21, and 23 (*p* < 0.05) (Figure 1A). As shown in Figure 1B, the body weight gain of the saline control group was higher than the LPS group and CGA + LPS group on days 15 and 17 (*p* < 0.05). Decreased feed intake was observed in the LPS group compared to control on days 15, 17 and 23 (*p* < 0.05). Inclusion of CGA with LPS injection increased feed intake at d15 (*p* < 0.05) and the feed intake of the CGA + LPS group was higher than the saline control group (*p* < 0.05) (Figure 1C). The highest FCR was noted for the CGA + LPS group compared to the control group with the LPS group being intermediate at d15 (*p* < 0.05). The control group had a lower FCR than the CGA + LPS group on days 17, 19, and 21(*p* < 0.05). The FCR of the CGA + LPS group was higher than the LPS group on d21 (*p* < 0.05) (Figure 1D). As shown in Figure 1E, the body weight of the control group was higher than the high-density + CGA group on days 7, 14, 21, and 28 (*p* < 0.05). High stocking density significantly decreased body weight on d28 in contrast to normal density control (*p* < 0.05) and the body weights of the high-density + CGA group was lower than the high-density group with no supplementation on d14 (*p* < 0.05). High stocking density significantly decreased body weight on days 1 to 7, 15 to 21, and 22 to 28 compared with the control group (*p* < 0.05) and the body weight gain of high-density + CGA animals was lower than controls on days 1 to 7, 8 to 14, and 22 to 28 (*p* < 0.05) (Figure 1F). Decreased feed intake was observed in the high-density group and the high-density + CGA group compared to the control group on days 8 to 14, 15 to 21, and 22 to 28 (*p* < 0.05). Inclusion of CGA increased feed intake on days 8 to 14 under high density stocking (*p* < 0.05) (Figure 1G). Broilers in the high-density + CGA group had a higher FCR than those in the control and high-density group at day 8 to 14 (*p* < 0.05). However, the FCR of the high-density + CGA group was lower than the control and high-density groups on days 15 to 21 (*p* < 0.05) (Figure 1H).

### 3.2. Serum Concentration of Corticosterone

**Figure 2:**
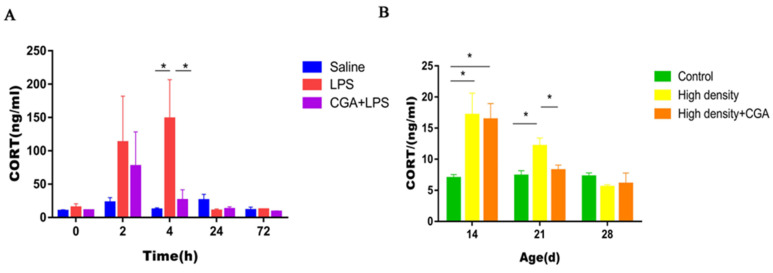
Effects of chlorogenic acid on serum corticosterone concentrations of broilers challenged with immune stress and high density stress. (**A**) Serum corticosterone concentrations after immune stress. (**B**) Serum corticosterone concentrations after high density stress. * means significant difference between groups.

The changes in the levels of corticosterone are shown in Figure 2. The corticosterone concentration in serum from the LPS group was increased at 4 h after LPS injection and CGA supplementation restored the CORT concentration to the initial level (*p* < 0.05) (Figure 2A). When compared to the control group, serum CORT concentrations in the high-density group increased significantly on days 14 and 21 (*p* < 0.05). CGA significantly decreased CORT concentrations on day 21 under high-density stocking (*p* < 0.05) (Figure 2B).

### 3.3. Serum Glutathione and Malondialdehyde Concentrations

**Figure 3:**
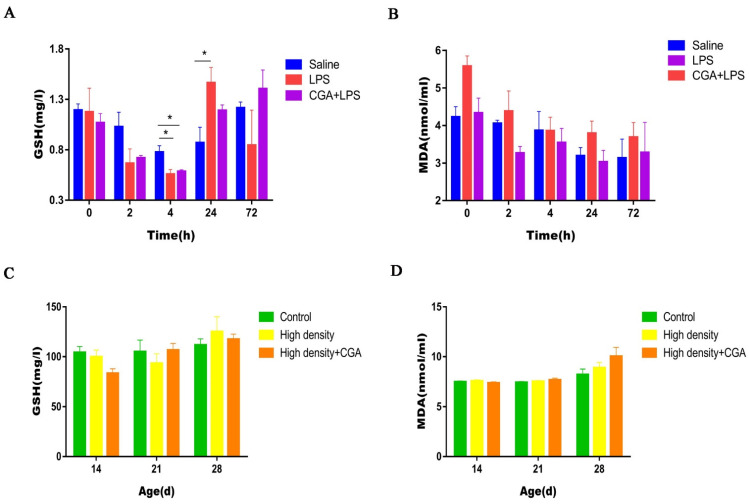
Effects of chlorogenic acid on serum glutathione and malondialdehyde levels of broilers challenged with immune stress and high density stress. (**A**) GSH levels of broilers challenged with immune stress. (**B**) MDA levels of broilers challenged with immune stress. (**C**) GSH levels of broilers challenged with high density stress. (**D**) MDA levels of broilers challenged with high density stress. GSH = glutathione, MDA = malondialdehyde, * means significant difference between groups.

Decreased concentrations of glutathione were observed in the LPS group and the CGA + LPS group compared to the saline control group at 4 h after LPS injection (*p* < 0.05). LPS injection significantly increased serum glutathione levels at 24 h after LPS injection (*p* < 0.05) (Figure 3A). Serum malondialdehyde concentrations were not affected by either LPS injection or CGA supplementation (*p* > 0.05) (Figure 3B). As shown in Figure 3C,D, serum glutathione and malondialdehyde concentrations were not affected by either high density or CGA supplementation (*p* > 0.05).

### 3.4. Intestinal Glutathione Concentrations

**Figure 4:**
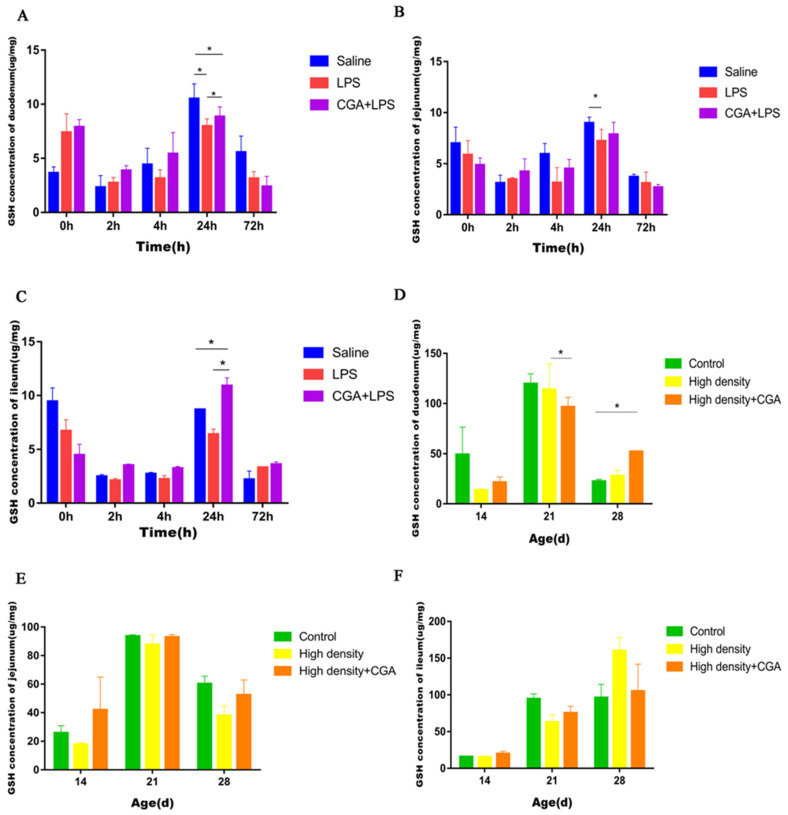
Effects of chlorogenic acid on intestinal glutathione levels of broilers challenged with immune stress and high density stress. (**A**) GSH levels of duodenum after immune stress. (**B**) GSH levels of jejunum after immune stress. (**C**) GSH levels of ileum after immune stress. (**D**) GSH levels of duodenum after high density stress. (**E**) GSH levels of jejunum after high density stress. (**F**) GSH levels of ileum challenged after high density stress. GSH = glutathione, * means significant difference between groups.

The highest glutathione concentration in the duodenum was noted for the saline control group compared to the LPS group, with the CGA + LPS group being intermediate at 24 h after LPS injection (*p* < 0.05) (Figure 4A). LPS significantly reduced jejunal glutathione concentration at 24 h after injection (*p* < 0.05) (Figure 4B). An increased glutathione concentration was also observed in the ileum in the CGA + LPS group compared to the control and LPS groups at 24 h after injection (*p* < 0.05) (Figure 4C). Figure 4D shows that the duodenal glutathione concentration of the high-density + CGA group was lower than the high-density group at day 21 (*p* < 0.05). The duodenal glutathione concentration in the high-density + CGA group was higher than the control group at day 28 (*p* < 0.05). Jejunal (Figure 4E) and ileal (Figure 4F) glutathione concentrations were not affected by either high stocking density or CGA supplementation (*p* > 0.05).

### 3.5. Serum Cytokine Concentration

**Figure 5:**
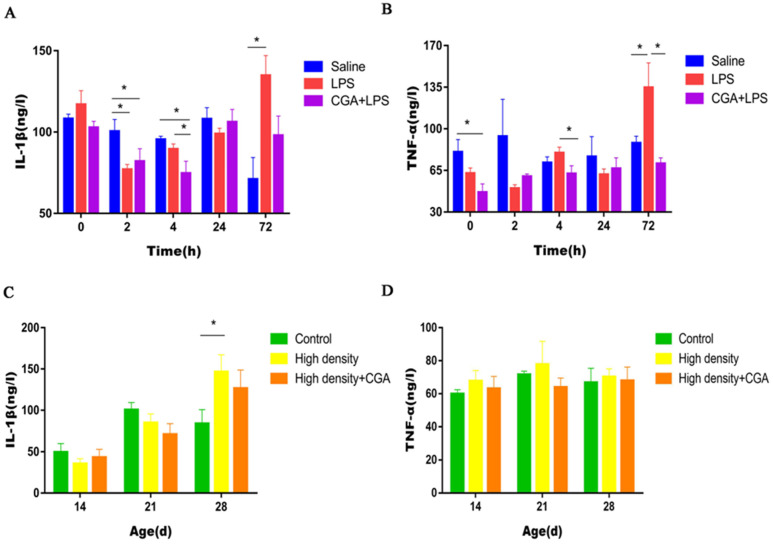
Effects of chlorogenic acid on serum cytokine levels of broilers challenged with immune stress and high density stress. (**A**) IL-1β levels of broilers challenged with immune stress. (**B**) TNF-α levels of broilers challenged with immune stress. (**C**) IL-1β levels of broilers challenged with high density stress. (**D**) TNF-α levels of broilers challenged with high density stress. IL = Interleukin, TNF-α = tumor necrosis factor-α, * means significant difference between groups.

As illustrated in Figure 5A, the LPS group and CGA + LPS group had lower serum IL-1β level in contrast to the saline control group 2 h after LPS injection (*p* < 0.05). In comparison between the control and LPS groups, the addition of CGA to the diet decreased the concentration of IL-1β 4 h after LPS injection (*p* < 0.05). LPS injection significantly increased serum IL-1β level at 72 h after LPS injection (*p* < 0.05). Dietary supplementation with CGA significantly reduced the concentration of TNF-α before LPS injection (*p* < 0.05). LPS significantly increased the serum TNF-α level at 72 h after LPS injection (*p* < 0.05), but inclusion of CGA decreased the concentration of TNF-α compared to the LPS group at 4 and 72 h after LPS injection (*p* < 0.05) (Figure 5B). High density stocking significantly increased the serum IL-1β level on day 28 (*p* < 0.05) (Figure 5C), but did not affect the serum TNF-α level (*p* > 0.05) (Figure 5D).

## 4. Discussion

Immune stress caused by LPS and stress from high stocking density are two major concerns in poultry production and these two stresses greatly impair the productive and reproductive performance of chickens with grave economic consequences. Studies have shown that compromised growth rate, feed intake, and FCR of chickens have been associated with immune stress and high-density stress [20,21,22,23]. Similarly, our results indicate that LPS injection and high stocking density significantly decrease body weight and feed the intake of broiler breeders. One primary cause of this effect could be that the hypothalamic-pituitary-adrenal axis is activated, and signal receptors related to appetite regulation are altered, leading to anorexia. Another possible explanation would be that pro-inflammatory cytokines are produced under immune stress, resulting in the repartition of nutrients and an increase in the catabolic activities associated with broiler growth [22,24].

In this study, our results revealed that CGA supplementation increased feed intake of broiler breeders stressed by LPS injection or high stocking density, suggesting that dietary CGA may alter signal receptors related to appetite regulation and thereby be beneficial in reducing stress-induced anorexia. CGA supplementation showed no effect on the body weight of broiler breeders either after the LPS challenge or housing under high stocking density. Therefore, the increased feed intake and unchanged body weight of stressed broiler breeders after CGA supplementation led to increased FCR in the early growth stage. Contrary to our findings, Zhao [11] reported that adding CGA extracted from *E. ulmoides* leads to an improved diet and the growth performance of heat-stressed broilers. Broiler chickens fed with CGA had a higher ADG than the dexamethasone-challenged animals [25]. Dietary CGA reduced F/G and increased the survival rate from *Clostridium perfringens* type A infection [26]. These studies indicated that the growth-promoting effects of CGA in chickens could be attributed not only to the strengthening of the intestinal mucosal barrier and immune function, but also to improving the ability to digest and absorb nutrients. The varying results of CGA on the growth performance of animals may be due to differences in animal health status, age, and species, as well as differences in the source or dosages of CGA.

The hypothalamopituitary-adrenocortical cascade is activated when broilers are exposed to stressors, resulting in corticosterone concentrations rising sharply [27]; thus ,the concentration of corticosterone provides a reliable indicator of a chicken’s stress status. Our results revealed that LPS injection and high stocking density each increased the concentration of corticosterone in serum, suggesting that the hypothalamic-pituitary-adrenal axis of broiler breeders was activated. We further evaluated the stress effects in this study by measuring the serum malondialdehyde concentration to assess the degree of peroxidation. The unchanged malondialdehyde concentration and increased corticosterone concentration in serum support the idea that LPS injection and high stocking density induced oxidative stress, but did not cause oxidative damage in broiler breeders. CGA remarkably downregulated serum corticosterone levels, alleviating the stress response of broiler breeders. Our results are in line with other studies showing that dietary CGA supplementation significantly decreased serum corticosterone concentration in DEX-challenged broilers [25].

Glutathione (GSH) is a critical antioxidant enzyme involved in the removal of radicals. GSH concentration was measured in broiler breeders after LPS challenge or stocking at high density to determine oxidative stress level and to assess the antioxidant activity of CGA. Our results are consistent with others’ findings that the GSH content decreases after LPS treatment [28,29], but in this study, changes in GSH under high stocking density were not observed. Because some research results showed that overcrowding induced oxidative stress in broilers, as represented by a decrease in GSH level in the liver [23], the variations in GSH level could be correlated with the different stocking densities we used or different species of broilers. In the present study, CGA increased the GSH content in the intestines of immune-stressed broiler breeders, in agreement with Shi [30], who found that CGA significantly increased GSH levels in liver tissue and reduced oxidative stress. Several studies have noted that CGA significantly enhanced antioxidant activity in broilers [11,12,25,26] and pigs [13,31], which was in agreement with our findings. The antioxidant effects of CGA were potentially attributed to its chemical structure for direct scavenging of free radicals and activation of endogenous antioxidant defenses against radicals [32].

Measuring cytokine production is an integral part of evaluating the cell-mediated immune response [33]. IL-1β, a major coordinator of the immune response, can stimulate immune cells to release a variety of cytokines [34]. TNF-α is a key cell signaling protein in the inflammatory response and induced apoptosis in a variety of cell types [35]. In our study, we found that LPS injection strongly promoted the production of circulating proinflammatory cytokines (IL-1β and TNF-α) in broiler breeders 72 h after LPS injection. Similarly, we observed that a high stocking density resulted in upregulated serum IL-1β levels after 28 days exposure. Dietary CGA restored the normal concentration of TNF-α in serum, which showed that CGA could at least partially counteract the LPS-induced inflammatory stress response. This is in agreement with the conclusions of many studies that CGA has anti-inflammatory activities [36,37]. Supplemental CGA in the feed exhibited significant anti-inflammatory effects by decreasing the expression of IL-1β and TNF-α in heat-stressed broilers [12], in broilers infected with *Clostridium perfringens* type A [26], and in IBV-infected chickens [38].

## 5. Conclusions

In summary, dietary CGA supplementation could increase the feed intake of broiler breeders and alleviate their stress response to LPS injection and high stocking density exposure. Therefore, CGA administration may be helpful in reducing anorexia in broiler breeders caused by stress. These findings imply that the possible roles of CGA are in preventing the inhibition of growth performance, anti-oxidant function, and immune response by immune stress and high stocking density stress, and providing broad implications for potential new strategies to counteract stress-induced impairments in broilers.

## Figures and Tables

**Table 1:** Composition and nutrient levels of the experimental basal diet, on an as-fed basis unless stated otherwise, %.

Items	1 to 28 Days
**Composition, %**	
Corn	60.1
Soybean meal	33.07
Soybean oil	3.6
Limestone-calcium carbonate	1.1
Calcium hydrogen phosphate	1
DL-Methionine, 98%	0.2
L-Lysine HCL, 78%	0.2
Sodium chloride	0.3
Vitamin Premix ^1^	0.03
Mineral Premix ^2^	0.2
Choline chloride, 50%	0.15
Ethoxyquin, 33%	0.05
Total	100
**Calculated Nutrient levels ^3^**	
Metabolizable energy, kcal/kg	2990
Crude protein, %	20.5
Calcium, %	0.99
Available phosphorus %	0.44
Lysine, %	1.1
Methionine, %	0.47

^1^ Vitamin premix provided the following per kg of diets: vitamin A (retinylacetate), 12,500 IU; vitamin D3 (cholecalciferol), 2500 IU; vitamin E (DL-a-tocopherol acetate), 18.75 mg; vitamin K3 (menadione sodium bisulfate), 2.65 mg; VB1 2 mg; VB2 6 mg; VB6 6 mg; vitamin B12 (cyanocobalamin), 0.025 mg; biotin, 0.0325 mg; folic acid, 1.25 mg; pantothenic acid, 1.25 mg; nicotinic acid, 50 mg. ^2^ Mineral premix provided per kilogram of complete diet: Cu (as copper sulfate) 8 mg, Zn (as zinc sulfate) 75 mg, Fe (as ferrous sulfate) 80 mg, Mn (as manganese sulfate) 100 mg, Se (as sodium selenite) 0.15 mg, I (as potassium iodide) 0.35 mg. ^3^ Calculated value based on the analyzed data of experimental diets.

## Data Availability

Not applicable.

## References

[1] Klasing K.C., Laurin D.E., Peng R.K., Fry D.M. (1987). Immunologically mediated growth depression in chicks: Influence of feed intake, corticosterone and interleukin-1. J. Nutr..

[2] Zhang X., Zhong X., Zhou Y., Wang G., Du H., Wang T. (2010). Dietary RRR-alpha-tocopherol succinate attenuates lipopolysaccharide-induced inflammatory cytokines secretion in broiler chicks. Br. J. Nutr..

[3] Star L., Kemp B., van den Anker I., Parmentier H.K. (2008). Effect of single or combined climatic and hygienic stress in four layer lines: 1. Performance. Poult. Sci..

[4] Yang X., Guo Y., He X., Yuan J., Yang Y., Wang Z. (2008). Growth performance and immune responses in chickens after challenge with lipopolysaccharide and modulation by dietary different oils. Animal.

[5] Lai H.T., Nieuwland M.G., Kemp B., Aarnink A.J., Parmentier H.K. (2011). Effects of repeated intratracheally administered lipopolysaccharide on primary and secondary specific antibody responses and on body weight gain of broilers. Poult. Sci..

[6] Jobe M.C., Ncobela C.N., Kunene N.W., Opoku A.R. (2019). Effects of Cassia abbreviata extract and stocking density on growth performance, oxidative stress and liver function of indigenous chickens. Trop. Anim. Health Prod..

[7] Li W., Wei F., Xu B., Sun Q., Deng W., Ma H., Bai J., Li S. (2019). Effect of stocking density and alpha-lipoic acid on the growth performance, physiological and oxidative stress and immune response of broilers. Asian-Australas. J. Anim. Sci..

[8] Suzuki A., Yamamoto N., Jokura H., Yamamoto M., Fujii A., Tokimitsu I., Saito I. (2006). Chlorogenic acid attenuates hypertension and improves endothelial function in spontaneously hypertensive rats. J. Hypertens..

[9] da Silveira T.F.F., Meinhart A.D., de Souza T.C.L., Cunha E.C.E., de Moraes M.R., Filho J.T., Godoy H.T. (2017). Optimization of the Preparation Conditions of Yerba Mate tea Beverage to Maximize Chlorogenic Acids Extraction. Plant Foods Hum. Nutr..

[10] Agunloye O.M., Oboh G., Ademiluyi A.O., Ademosun A.O., Akindahunsi A.A., Oyagbemi A.A., Omobowale T.O., Ajibade T.O., Adedapo A.A. (2019). Cardio-protective and antioxidant properties of caffeic acid and chlorogenic acid: Mechanistic role of angiotensin converting enzyme, cholinesterase and arginase activities in cyclosporine induced hypertensive rats. Biomed. Pharmacother..

[11] Zhao J.S., Deng W., Liu H.W. (2019). Effects of chlorogenic acid-enriched extract from Eucommia ulmoides leaf on performance, meat quality, oxidative stability, and fatty acid profile of meat in heat-stressed broilers. Poult. Sci..

[12] Chen F., Zhang H., Zhao N., Yang X., Du E., Huang S., Guo W., Zhang W., Wei J. (2021). Effect of chlorogenic acid on intestinal inflammation, antioxidant status, and microbial community of young hens challenged with acute heat stress. Anim. Sci. J..

[13] Chen J., Yu B., Chen D., Huang Z., Mao X., Zheng P., Yu J., Luo J., He J. (2018). Chlorogenic acid improves intestinal barrier functions by suppressing mucosa inflammation and improving antioxidant capacity in weaned pigs. J. Nutr. Biochem..

[14] Liang N., Kitts D.D. (2018). Amelioration of Oxidative Stress in Caco-2 Cells Treated with Pro-inflammatory Proteins by Chlorogenic Acid Isomers via Activation of the Nrf2-Keap1-ARE-Signaling Pathway. J. Agric. Food Chem..

[15] Vukelic I., Detel D., Pucar L.B., Potocnjak I., Buljevic S., Domitrovic R. (2018). Chlorogenic acid ameliorates experimental colitis in mice by suppressing signaling pathways involved in inflammatory response and apoptosis. Food Chem. Toxicol..

[16] Wang L., Bi C., Cai H., Liu B., Zhong X., Deng X., Wang T., Xiang H., Niu X., Wang D. (2015). The therapeutic effect of chlorogenic acid against Staphylococcus aureus infection through sortase A inhibition. Front. Microbiol..

[17] Gong X.X., Su X.S., Zhan K., Zhao G.Q. (2018). The protective effect of chlorogenic acid on bovine mammary epithelial cells and neutrophil function. J. Dairy Sci..

[18] Wang G.F., Shi L.P., Ren Y.D., Liu Q.F., Liu H.F., Zhang R.J., Li Z., Zhu F.H., He P.L., Tang W. (2009). Anti-hepatitis B virus activity of chlorogenic acid, quinic acid and caffeic acid in vivo and in vitro. Antivir. Res..

[19] Shin H.S., Satsu H., Bae M.J., Zhao Z., Ogiwara H., Totsuka M., Shimizu M. (2015). Anti-inflammatory effect of chlorogenic acid on the IL-8 production in Caco-2 cells and the dextran sulphate sodium-induced colitis symptoms in C57BL/6 mice. Food Chem..

[20] Yang X.J., Li W.L., Feng Y., Yao J.H. (2011). Effects of immune stress on growth performance, immunity, and cecal microflora in chickens. Poult. Sci..

[21] Goo D., Kim J.H., Choi H.S., Park G.H., Han G.P., Kil D.Y. (2019). Effect of stocking density and sex on growth performance, meat quality, and intestinal barrier function in broiler chickens. Poult. Sci..

[22] Yang S., Zhang J., Jiang Y., Xu Y.Q., Jin X., Yan S.M., Shi B.L. (2021). Effects of Artemisia argyi flavonoids on growth performance and immune function in broilers challenged with lipopolysaccharide. Anim. Biosci..

[23] Sugiharto S. (2022). Dietary strategies to alleviate high-stocking-density-induced stress in broiler chickens—A comprehensive review. Arch. Anim. Breed..

[24] Zheng X.C., Wu Q.J., Song Z.H., Zhang H., Zhang J.F., Zhang L.L., Zhang T.Y., Wang C., Wang T. (2016). Effects of Oridonin on growth performance and oxidative stress in broilers challenged with lipopolysaccharide. Poult. Sci..

[25] Zhang K., Li X., Zhao J., Wang Y., Hao X., Liu K., Liu H. (2022). Protective effects of chlorogenic acid on the meat quality of oxidatively stressed broilers revealed by integrated metabolomics and antioxidant analysis. Food Funct..

[26] Zhang X., Zhao Q., Ci X., Chen S., Xie Z., Li H., Zhang H., Chen F., Xie Q. (2020). Evaluation of the efficacy of chlorogenic acid in reducing small intestine injury, oxidative stress, and inflammation in chickens challenged with Clostridium perfringens type A. Poult. Sci..

[27] Weimer S.L., Wideman R.F., Scanes C.G., Mauromoustakos A., Christensen K.D., Vizzier-Thaxton Y. (2018). An evaluation of methods for measuring stress in broiler chickens. Poult. Sci..

[28] Kheir-Eldin A.A., Motawi T.K., Gad M.Z., Abd-ElGawad H.M. (2001). Protective effect of vitamin E, beta-carotene and N-acetylcysteine from the brain oxidative stress induced in rats by lipopolysaccharide. Int. J. Biochem. Cell Biol..

[29] Gou Z., Jiang S., Zheng C., Tian Z., Lin X. (2015). Equol Inhibits LPS-Induced Oxidative Stress and Enhances the Immune Response in Chicken HD11 Macrophages. Cell. Physiol. Biochem..

[30] Shi A., Shi H., Wang Y., Liu X., Cheng Y., Li H., Zhao H., Wang S., Dong L. (2018). Activation of Nrf2 pathway and inhibition of NLRP3 inflammasome activation contribute to the protective effect of chlorogenic acid on acute liver injury. Int. Immunopharmacol..

[31] Zhang Y., Wang Y., Chen D., Yu B., Zheng P., Mao X., Luo Y., Li Y., He J. (2018). Dietary chlorogenic acid supplementation affects gut morphology, antioxidant capacity and intestinal selected bacterial populations in weaned piglets. Food Funct..

[32] Liang N., Kitts D.D. (2015). Role of Chlorogenic Acids in Controlling Oxidative and Inflammatory Stress Conditions. Nutrients.

[33] Zhen W., Shao Y., Wu Y., Li L., Pham V.H., Abbas W., Wan Z., Guo Y., Wang Z. (2020). Dietary yeast beta-glucan supplementation improves eggshell color and fertile eggs hatchability as well as enhances immune functions in breeder laying hens. Int. J. Biol. Macromol..

[34] Gu T., Li G., Wu X., Zeng T., Xu Q., Li L., Vladyslav S., Chen G., Lu L. (2020). Effects of immunopotentiators on biochemical parameters, proinflammatory cytokine, and nonspecific immune responses in Shaoxing ducklings. Poult. Sci..

[35] Gu T., Lu L., Xu W., Zeng T., Tian Y., Chen B., Chen L., Shen J., Li G. (2022). Immunopotentiators improve the antioxidant defense, apoptosis, and immune response in Shaoxing ducklings. Poult. Sci..

[36] dos Santos M.D., Almeida M.C., Lopes N.P., de Souza G.E. (2006). Evaluation of the anti-inflammatory, analgesic and antipyretic activities of the natural polyphenol chlorogenic acid. Biol. Pharm. Bull..

[37] Hwang S.J., Kim Y.W., Park Y., Lee H.J., Kim K.W. (2014). Anti-inflammatory effects of chlorogenic acid in lipopolysaccharide-stimulated RAW 264.7 cells. Inflamm. Res..

[38] Abaidullah M., Peng S., Song X., Zou Y., Li L., Jia R., Yin Z. (2021). Chlorogenic acid is a positive regulator of MDA5, TLR7 and NF-kappaB signaling pathways mediated antiviral responses against Gammacoronavirus infection. Int. Immunopharmacol..

